# Classification and Prediction of Violence Against Chinese Medical Staff on the Sina Microblog Based on a Self-Organizing Map: Quantitative Study

**DOI:** 10.2196/13294

**Published:** 2020-05-26

**Authors:** Guimin Duan, Xin Liao, Weiping Yu, Guihua Li

**Affiliations:** 1 School of Management Chengdu University of Traditional Chinese Medicine Chengdu, Sichuan China; 2 School of Public Affairs and Law Southwest Jiaotong University Chengdu, Sichuan China; 3 School of Business Sichuan University Chengdu, Sichuan China; 4 School of Public Administration Sichuan University Chengdu, Sichuan China

**Keywords:** workplace violence, medical staff, social media

## Abstract

**Background:**

For the last decade, doctor-patient contradiction in China has remained prominent, and workplace violence toward medical staff still occurs frequently. However, little is known about the types and laws of propagation of violence against medical staff online.

**Objective:**

By using a self-organizing map (SOM), we aimed to explore the microblog propagation law for violent incidents in China that involve medical staff, to classify the types of incidents and provide a basis for rapidly and accurately predicting trends in public opinion and developing corresponding measures to improve the relationship between doctors and patients.

**Methods:**

For this study, we selected 60 cases of violent incidents in China involving medical staff that led to heated discussions on the Sina microblog from 2011 to 2018, searched the web data of the microblog using crawler software, recorded the amount of new tweets every 2 hours, and used the SOM neural network to cluster the number of tweets. Polynomial and exponential functions in MATLAB software were applied to predict and analyze the data.

**Results:**

Trends in the propagation of online public opinion regarding the violent incidents were categorized into 8 types: bluff, waterfall, zigzag, steep, abrupt, wave, steep slope, and long slope. The communications exhibited different characteristics. The prediction effect of 4 types of incidents (ie, bluff, waterfall, zigzag, and steep slope) was good and accorded with actual spreading trends.

**Conclusions:**

Our study found that the more serious the consequences of a violent incident, such as a serious injury or death, the more attention it drew on the microblog, the faster was its propagation speed, and the longer was its duration. In these cases, the propagation types were mostly steep slope, long slope, and zigzag. In addition, the more serious the consequences of a violent incident, the higher popularity it exhibited on the microblog. The popularity within a week was significantly higher for acts resulting from patients’ dissatisfaction with treatments than for acts resulting from nontherapeutic incidents.

## Introduction

### Background

At the beginning of 2009, China started a new round of medical and health system reformation. Its guiding ideology was to continuously improve the health level of the whole population and promote social harmony, which has improved, to a great extent, the doctor-patient relationship. However, contradictions between doctors and patients are still prominent, and workplace violence toward medical staff occur frequently. The Chinese Hospital Association reported that the proportion of hospitals experiencing workplace violence increased by 90% from 2008 to 2012 [1]. In 2017 itself, there were as many as 54 violent incidents against Chinese medical staff mentioned on the Sina microblog (a leading microblog website and one of the largest social media platforms in China), which attracted public attention and sparked heated debate. For instance, on December 8, 2017, in Tongshan County People’s Hospital, a patient’s mother hurt a nurse because she was not satisfied with the treatment effect, causing slight bodily injury (signs of abortion) to the medical staff. When the incident was publicized on the Sina microblog, the number of tweets reached 1737 within a week. The frequent outbreak of violent incidents in hospitals and the wide coverage of the incidents by social media have made the doctor-patient relationship even more fraught, which not only reduces the professional security of medical staff [2] but also hinders their professional performance, resulting in a negative impact on patient service and overall health [3-8]. Moreover, violent incidents have a negative impact on the psychological welfare of health care workers who do not want their children to be engaged in health care [9-11].

Workplace violence in the health sector is a worldwide concern, with health care workers being at high risk of being victims [12]. Following the World Health Organization’s definition, workplace violence takes 2 main forms: physical and nonphysical violence. Physical violence includes hitting, slapping, kicking, pushing, choking, grabbing, sexual assault, and other forms of physical contact intended to injure or harm. In contrast, nonphysical violence includes threats, sexual harassment, bullying, and verbal abuse and may be perpetrated by various types of people [13]. There are 2 major sources of workplace violence: coworker initiated and public initiated [14]. In the Chinese health sector, workplace violence is mainly caused by patients and their relatives or friends. Scholars have studied the causes of violent incidents from various perspectives. On the basis of the perspective of social roles, Robinson [15] pointed out that due to the differences in the roles of doctors and patients, their viewpoints and interests differ, which leads to cognitive and motivational bias in the attribution process regarding medical information. From the point of view of social psychology, Feng et al [4] analyzed the mechanisms for the inducement of violent incidents related to doctor-patient conflicts, including psychological frustration, anger, social learning, and so on. From the perspective of patient experience, Hu et al [5] proposed that the experience of a poor physical environment, inadequate information communication, large medical expenses, and the perception and experience of treatment outcomes were direct causes of doctor-patient contradiction. From the perspective of crisis management, Li [6] posited that it was caused by conflicts of interest, the inadequate communication of information, negative slants in public opinion, and so on. Duan et al [16] used content analysis to classify violent incidents and associated them with 6 factors: diagnosis and treatment effects, doctor-patient communication, response speed, medical expenses, privacy protection, and patients’ or their families’ problems.

### Objectives

With the development of social media, the microblog has become a key vehicle of public opinion by virtue of its extensive and real-time capabilities for mass communication. Despite the widespread coverage of violent incidents toward medical staff on social media, little empirical research has examined their types and laws of propagation. Although scholars have studied public opinion about violent incidents, they have focused mainly on influencing factors, the dynamic mechanisms of public opinion, and the characteristics of the incidents [17,18]. Some scholars have conducted research on the types and characteristics of microblog communication in light of corporate crisis events [19]. However, due to the particularity of medical services and the diversity of incentives for committing violent acts against medical staff, existing research results cannot effectively guide hospitals and health management departments in managing public opinion about the incidents. In relation to this issue, we took violent incidents against Chinese medical staff as the research object and applied two methods—the self-organizing map (SOM) neural network and function fitting—to conduct a quantitative study. The purpose of this study was to explore the microblog propagation law for violent incidents in China that involve medical staff, to classify the types of incidents, and to provide a basis for rapidly and accurately predicting trends in public opinion and developing corresponding measures to improve the relationship between doctors and patients. We selected 60 cases of violent incidents that led to heated discussions on the Sina microblog from 2011 to 2018 and used crawler software to search the microblog’s web data. Starting from the first post about the incidents, every 2 hours, we recorded the number of additional tweets. We used the SOM to demarcate changes in the number of tweets and used the exponential and polynomial functions in MATLAB R2017a (The MathWorks) to predict and analyze the data to achieve better prediction. For hospitals and health management departments, understanding the communication types and the characteristics of the violent incidents being discussed on microblogs such as the Sina microblog can be helpful to institutions for scientifically predicting the propagation rules of incidents, for effectively managing and guiding public opinion relating to doctors and patients, and for improving the doctor-patient relationship.

## Methods

### Data Collection and Processing

We used “doctor/nurse was hit,” “doctor/nurse was slapped,” “doctor/nurse was killed,” “doctor/nurse was attacked,” “doctor/nurse was hurt,” and so on as keywords when searching for violent incidents from January 1, 2011, to July 31, 2018, using the Baidu search engine, and we searched for complete data on the development and outcomes of the target incidents. In total, we collected 403 violent incidents involving Chinese medical staff. According to Duan’s classification criteria, we used the content analysis method to analyze the inducements for 60 violent incidents and grouped them into 6 categories: treatment effect, doctor-patient communication, response speed, medical expenses, privacy protection, and patients’ or families’ own problems [16]. Relying on the crawler software platform of GooSeeker (Shenzhen Tianju Information Technology Ltd), we defined the fetching rules, searched related tweets by keyword, and then collected the website data from the Sina microblog to record the total number of tweets in a week.

As the propagation characteristics of violent incidents against Chinese medical staff on the Sina microblog are not obvious and the degree of influence is relatively low, we considered the total number of tweets of violent incidents on the Sina microblog within a week as the standard and selected 60 typical incidents that drew heated responses and discussion among netizens from 2011 to 2018 as the study samples. The samples covered the 5 direct causes of violent incidents involving medical staff and were representative to some extent. In accordance with the “criteria for human injury evaluation” issued by the Judicial Department in China, the severity of the consequences of violent incidents was classified as “slight bodily injury,” “minor injury,” “serious injury,” “death,” or “serious influence on hospital order” (as listed in Multimedia Appendix 1).

We mainly studied the dynamics of public opinion in the first week after the violent incidents. Therefore, we started to record tweets about the first violent incident reports and then recorded newly added Sina microblog material every 2 hours. We searched 168 hours of data within a week and collected 84 datasets in total. There were differences in the number of tweets for different events, so the data were standardized.

The formula was as follows: adjusted standard value=100×(original value−min)/(max−min). Min and max are the minimum and maximum values for each of the 84 datasets about the incidents.

### Network Sample Design

**Figure 1:**
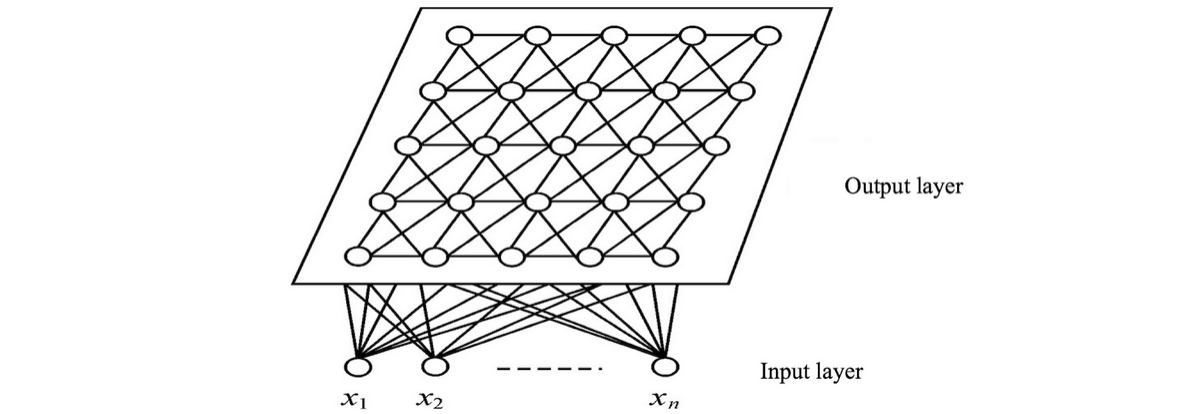
A topology diagram of the self-organizing map network.

The SOM is an excellent tool in the exploratory phase of data mining [20]. SOM networks learn to cluster groups of similar input patterns from a high-dimensional input space in a nonlinear fashion to a low-dimensional (most commonly two-dimensional) discrete lattice of neurons in an output layer [21], which was widely applied to classification and prediction [22-27]. The neurons in the competition layer can be one-dimensional, two-dimensional, or multidimensional (as shown in Figure 1 [28]). SOMs are reported to be robust and accurate with noisy data [29].

The SOM method works as follows [30]. Initially, one has to choose the topology of the map. All the nodes are linked to the input nodes by weighted edges. The weights are first set at random and then iteratively adjusted. Each iteration involves randomly selecting an object x and moving the closest node (and its neighborhood) in the direction of x. The closest node is obtained by measuring the Euclidean distance or the dot product between the object x and the weights of all nodes in the map. The neighborhood to be adjusted is defined by a neighborhood function, which decreases over time.

We used the MATLAB programming language to construct the SOM neural network model and employed functions provided by a neural network toolbox to implement the whole learning process, such as training, emulating functions, and so on. According to the data after standardized processing, the input mode of the network was determined as


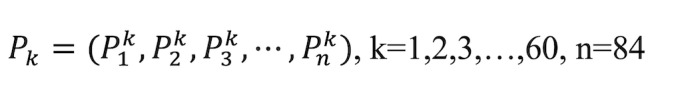


where k=1,2,3,…,60 (n=84), that is, there were 60 sets of samples, each of which contained 84 elements.

## Results

### Clustering Results

**Figure 2:**
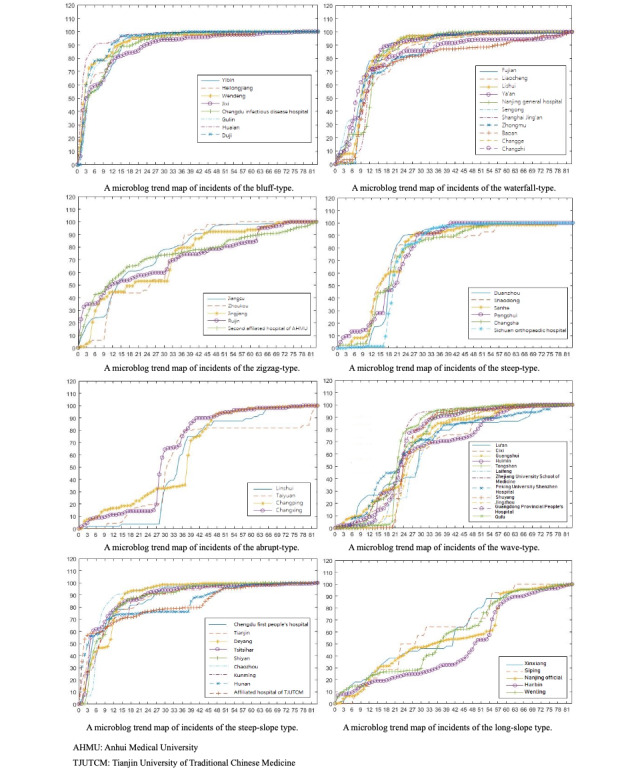
A microblog trend map of incidents of the types.

With the SOM neural network toolkit of MATLAB R2017a, 60 groups of data, containing 84 elements each, were clustered by standardized processing into 8 types. On the basis of the shapes of the trend lines in the graphs, the types of clustering results were named as follows: *bluff*, *waterfall*, *zigzag*, *steep*, *abrupt*, *wave*, *steep slope*, and *long slope* (shown in Figure 2).

By the application of the SOM neural network algorithm, 60 medical incidents against Chinese medical staff were divided into 8 types, based on the number of dynamic changes in the Sina microblog tweets. In Figure 2, it can be seen that these 8 types of curves generally showed significant differences, albeit similar curves represent similar trends. The types were all named according to the general shapes of the curves. It can be seen from the figures that the microblog transmission curves of each type had distinguishing characteristics (shown in Table 1). The clustering results of the SOM neural network model were good.

**Table 1:** Clustering results and curve characteristics.

Category	Hot violent incidents with medical staff	Curve characteristics
Bluff type	Yibin Second People’s Hospital, December 17, 2017; Heilongjiang Provincial People’s Hospital, August 15, 2017; Shandong Wendeng Osteopathic Hospital, March 21, 2017; Jixi People’s Hospital, July 11, 2017; Chengdu Infectious Disease Hospital, July 21, 2017; Gulin County People’s Hospital, January 14, 2015; Huai’an First People’s Hospital, February 10, 2018; Du Ji people’s Hospital, March 21, 2016	In the first 12 hours or so, there was a peak period of tweet postings. After which, in the 13th hour, the growth rate suddenly declined and then lasted for 6 hours. After that, in the 19th hour, the number of tweets sped up, and the increase lasted for 6 hours or so. Then, it continued to increase at a slower pace. After the 96th hour, the growth rate was almost zero.
Waterfall type	Fujian Provincial Cancer Hospital, February 7, 2017; Liaocheng People’s Hospital, December 4, 2017; Hong Lan Town Health Centre of Lishui District, April 23, 2017; Ya’an People’s Hospital, January 5, 2017; Nanjing General Hospital of Nanjing Command, January 17, 2017; Shanxi Sengong Hospital, February 1, 2017; Jing’an District Central Hospital, March 4, 2016; Yanminghu Central Health Center of Zhongmu County, June 11, 2015; Baoan District people’s Hospital, September 9, 2013; Changge People’s Hospital, November 1, 2015; Heping Hospital affiliated to Changzhi Medical College, November 22, 2016	The early growth rate was slow. At around 12 hours, the growth rate of the microblog increased sharply and then gradually slowed down.
Zigzag type	Jiangsu Provincial People’s Hospital, February 16, 2017; Zhoukou Central Hospital, July 12, 2017; Orthopaedic Clinic in Jingjiang City, April 22, 2017; Ruijin Hospital Affiliated to Medical College of Shanghai Jiaotong University, June 27, 2015; Second Affiliated Hospital of Anhui Medical University, November 13, 2012	Growth was rapid in the early stage, and then the growth trend became slow, or even close to 0, followed by a growth phase featuring alternating speeds.
Steep type	Women and Children Health Hospital of Duanzhou District, August 6, 2017; Shaodong County People’s Hospital, July 17, 2017; Sanhe People’s Hospital, March 21, 2017; Pengshui County People’s Hospital, March 24, 2017; Jiangbei Town Center Hospital of Changsha County, May 17, 2016; Sichuan Orthopaedic Hospital, October 10, 2016	In the early stages, the blog was in an incubation period. Then, there was a peak period of tweets posted between the 24th and 48th hours, after which the growth gradually slowed down. After the 114th hour, the growth rate was almost 0.
Abrupt type	Traditional Chinese Medicine Hospital of Linshui County, August 5, 2017; Taiyuan Central Hospital, August 5, 2017; Dongguan Changping Hospital, April 17, 2016; Changxing County People’s Hospital, October 4, 2016	The growth rate was slow in the first 30 hours or so, and then, it increased slightly over the next 2 hours or so. Before the 54th hour, the growth rate was almost flat. Then, a peak period of posting occurred between 54 and 72 hours, after which the growth rate gradually slowed down.
Wave type	Lu‘an People’s Hospital, September 1, 2017; Cixi people’s Hospital, June 4, 2017; Guangshui First People’s Hospital, April 6, 2017; Huimin People’s Hospital, June 15, 2017; Tongshan County People’s Hospital, December 8, 2017; Lai Feng County Central Hospital, November 2, 2017; The Second Affiliated Hospital of Zhejiang University School of Medicine, February 20, 2014; Peking University Shenzhen Hospital, February 25, 2014; Shuyang County Nanguan Hospital, April 19, 2014; Jingzhou First People’s Hospital, May 5, 2012; Guangdong Provincial People’s Hospital, May 5, 2016; Qufu People’s Hospital, May 16, 2016	The number of tweets increased gradually over the first 42 hours and then increased suddenly. Then, the growth rate showed a wave-shaped oscillation within 43 to 149 hours. After the 150th hour, the growth rate was almost 0.
Steep slope type	First People’s Hospital of Chengdu, March 23, 2017; Tianjin Third Central Hospital, June 29, 2017; Deyang People’s Hospital, January 3, 2016; Beigang Hospital, February 17, 2014; Shiyan People’s Hospital, February 21, 2015; Chaozhou Central Hospital, March 5, 2014; First Affiliated Hospital of Kunming Medical University, June 7, 2015; Hunan Academy of Traditional Chinese Medicine Affiliated Hospital, September 23, 2013; First Teaching Hospital of Tianjin University of Traditional Chinese Medicine, November 29, 2012	The number of tweets increased sharply over the first 6 hours or so. Then, there was a short pause, followed by a gradual and steady increase from the 84th hour to the 101st hour. After the 102nd hour, the growth rate was almost 0.
Long slope type	Xinxiang Second People’s Hospital, November 7, 2017; Siping Central Hospital, October 15, 2017; Nanjing Stomatological Hospital, February 25, 2014; First Affiliated Hospital of Harbin Medical University, March 23, 2012; Wenling First People’s Hospital, October 25, 2013	The growth rate of the tweets was stable and lasted for a long time. After 120 hours, it gradually increased.

### Correlation Analysis Between the Types of Propagation on the Microblog and the Characteristics of the Medical Incidents

The characteristics of the medical incidents against Chinese staff analyzed in this study were the severity of the consequences and the inducements. To explore the relationships between the types of propagation on the microblog and the severity of the consequences of the medical incidents, we conducted a one-way analysis of variance (ANOVA; single factor) considering the types of propagation on the microblog as the independent variables and severity of the consequence of the medical incidents as the dependent variable. After administering the Shapiro-Wilk test, the dependent variable was normally distributed. The results show that there were significant differences in the severity of the consequences of incidents associated with the different types of propagation (*P*=.03). The severity of the consequences of medical incidents of the long slope type (mean 4.00) was significantly higher than those of the bluff type (mean 2.63), waterfall type (mean 2.73), steep type (mean 2.17), abrupt type (mean 2.5), and wave type (mean 2.58). The severity of the consequences of medical incidents of the steep slope type (mean 3.75) was significantly higher than those of the bluff type (mean 2.63), steep type (mean 2.17), and wave type (mean 2.58). The severity of the consequences of medical incidents of the zigzag type (mean 3.80) was significantly higher than those of the steep type (mean 2.17) and wave type (mean 2.58), as shown in Multimedia Appendix 2.

The abovementioned analysis shows that the severity of the consequences of the medical incidents varies with the type of propagation. The severity of incidents of the long slope type, steep slope type, and zigzag type was higher than that of the other types, and most of the incidents in these categories involved a serious injury or death. Therefore, we classified these incidents as a high-severity group and the other incidents as a low-severity group. We used a one-way ANOVA, with these 2 groups, that characterized the consequences of the medical incidents as the independent variable and popularity within a week of the incidents as the dependent variable. Popularity was rated on a 6-point scale according to the number of tweets within a week. We coded 6 for a number of tweets above 5000, 5 for a number from 2000 to 5000, 4 for 1000 to 2000, 3 for 500 to 1000, 2 for 100 to 500, and 1 for 0 to 100. The higher the point score, the higher the popularity. Therefore, the dependent variable is continuous. After administering the Shapiro-Wilk test, the dependent variable was normally distributed. The results show that the popularity of high-severity incidents was significantly higher than that of low-severity incidents (high: mean 3.44; low: mean 2.05; *P*<.001). We adopted a one-way ANOVA, with the types of inducement for the incidents as independent variables and popularity within a week as the dependent variable. We found that violent acts resulting from patients’ dissatisfaction with treatment effects garnered popularity within a week that was significantly higher than the popularity of incidents caused by nontherapeutic effects (treatment effect: mean 3.05; nontherapeutic effect: mean 2.15; *P*=.02).

We constructed a cross-tabulation of the inducements of the medical incidents and the types of propagation on the microblog. The chi-square test results show that there was no correlation between them.

### Forecasting Trends in Network Public Opinion Regarding Violent Incidents

Nowadays, microblogs are becoming important carriers of public opinion on the internet about violent incidents against medical staff, and the traditional methods that medical institutions and health management departments use to control public opinion are severely weakened. The dynamic monitoring of trends and changes in microblog discussions can help hospital and health management departments to take timely countermeasures after violent incidents are publicized. At the same time, relevant departments could make use of the data to predict the propagation of microblog discussions of violent incidents, effectively manage and guide public opinion about doctors and patients, and improve the relationship between doctors and patients. On the basis of the study on stock price prediction by Afolabi and Olude [31], we considered 4 types of propagation, including the bluff, waterfall, zigzag, and steep slope types, for the prediction analysis in this study.

#### Forecasting Trends in the Spread of Network Public Opinion: Bluff-Type Propagation

**Figure 3:**
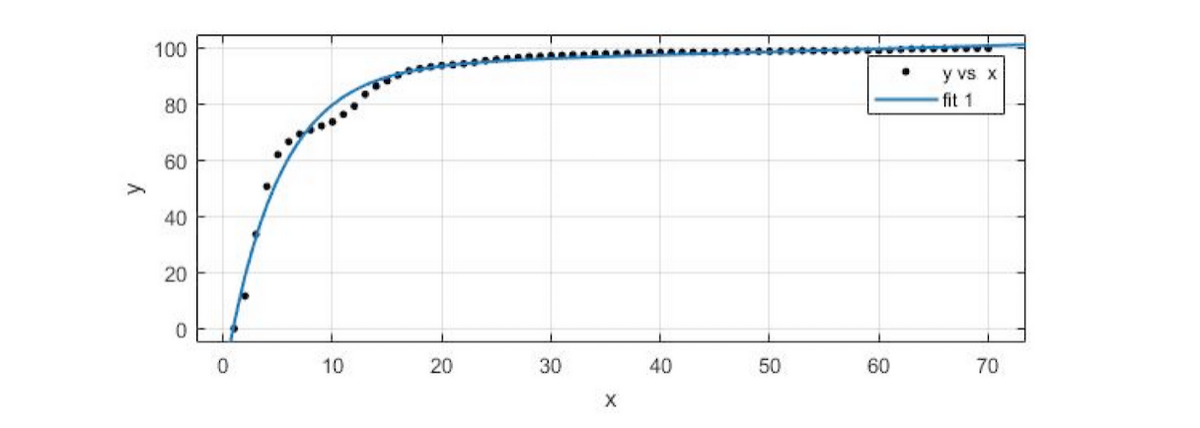
A data-fitting diagram of the bluff-type incidents.

The first cluster, consisting of incidents with the bluff-type profile, was selected for the sample. We took the average data of the 8 incidents in the Sina microblog and used the cftool toolkit in MATLAB R2017a to do the exponential function fitting for prediction. We recorded newly added Sina microblog material every 2 hours and searched 168 hours of data within a week, collecting 84 datasets in total. The first 70 of the 84 datasets were selected for fitting. We determined the fitting model and used the last 14 datasets as the prediction results to validate the data. As shown in Figure 3, *fit 1* was the fitting curve, and *y* vs *x* was the original data point.

The fitting model for the exponential function was

*f*(*x_1_*) = 93.39 × exp(0.001092 × *x_1_*)−112.8 × exp(−0.204 × *x_1_*)

where *x_1_* was the data observation point (we took observations every 2 hours) and *f*(*x_1_*) was the number of related tweets after standardized processing under the observation point.

In the fitting of the model, *R^2^* (the coefficient of determination) was 0.985, the sum of squares due to error (SSE) was 377.7, adjusted *R^2^* was 0.9846, and the root-mean-square error (RMSE) was 2.392.

As shown in Table 2, the predicted data values were almost consistent with the actual values, with a maximum relative error of 1.9055% and a minimum relative error of 0.77%. The trend of the data in the forecast section (positions 71 to 84) completely coincided with the actual trend, which shows that the prediction effect of the model for the bluff-type incidents was good.

**Table 2:** A comparison of the fitting value with the actual value of the bluff-type incidents.

Predicted position	Fitting value	Actual value	Relative error (%)
71	100.5967	99.8280	0.7700
72	100.6968	99.8590	0.8390
73	100.7970	99.8918	0.9062
74	100.8973	99.9188	0.9793
75	100.9976	99.9188	1.0796
76	101.0981	99.9729	1.1255
77	101.1987	99.9729	1.2261
78	101.2994	99.9729	1.3268
79	101.4001	100.0000	1.4001
80	101.5010	100.0000	1.5010
81	101.6020	100.0000	1.6020
82	101.7031	100.0000	1.7031
83	101.8042	100.0000	1.8042
84	101.9055	100.0000	1.9055

#### Forecasting Trends in the Spread of Network Public Opinion: Waterfall-Type Propagation

**Figure 4:**
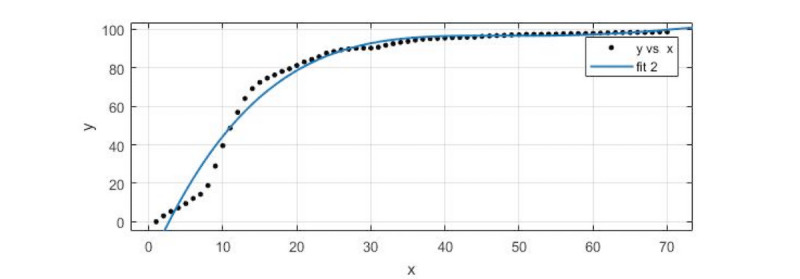
A data-fitting diagram of the waterfall-type incidents.

The second cluster, consisting of waterfall-type incidents, was selected for the sample. We took the average data of the 11 incidents in the Sina microblog for the forecast and made use of the cftool toolkit in MATLAB R2017a to do the polynomial function fitting for prediction. As shown in Figure 4, *fit 2* was the fitting curve.

The fitting model of the polynomial function was

*f*(*x_2_*) = (−1.156e-05)*x_2_*^4^ + 0.002763*x_2_*^3^−0.2373*x_2_*^2^ + 8.783*x_2_*−22.33

where *x_2_* was the data observation point (we made an observation every 2 hours) and *f*(*x_2_*) was the value of the number of related tweets after standardized processing under the observation point.

In the fitting of the model, *R^2^* was 0.977, the SSE was 1356, adjusted *R^2^* was 0.9756, and RMSE was 4.568.

As shown in Table 3, the predicted data values were almost consistent with the actual values, with a maximum relative error of 3.06% and a minimum relative error of 1.3401%. The trend of the data in the forecast section (positions 71 to 84) completely coincided with the actual trend, which shows that the prediction effect of the model for the waterfall-type incidents was good.

**Table 3:** A comparison of the fitting value with the actual value of the waterfall-type incidents.

Predicted position	Fitting value	Actual value	Relative error (%)
71	100.1828	98.8402	1.3401
72	100.5052	98.9306	1.5666
73	100.8276	99.0648	1.7483
74	101.1463	99.1315	1.9919
75	101.4575	99.2729	2.1532
76	101.7571	99.2850	2.4294
77	102.0408	99.2850	2.7007
78	102.3039	99.3088	2.9277
79	102.5415	99.4037	3.0600
80	102.7484	99.9145	2.7581
81	102.9191	99.9609	2.8743
82	103.0478	99.9609	2.9956
83	103.1286	100.0000	3.0337
84	103.1551	100.0000	3.0586

#### Forecasting Trends in the Spread of Network Public Opinion: Zigzag-Type Propagation

**Figure 5:**
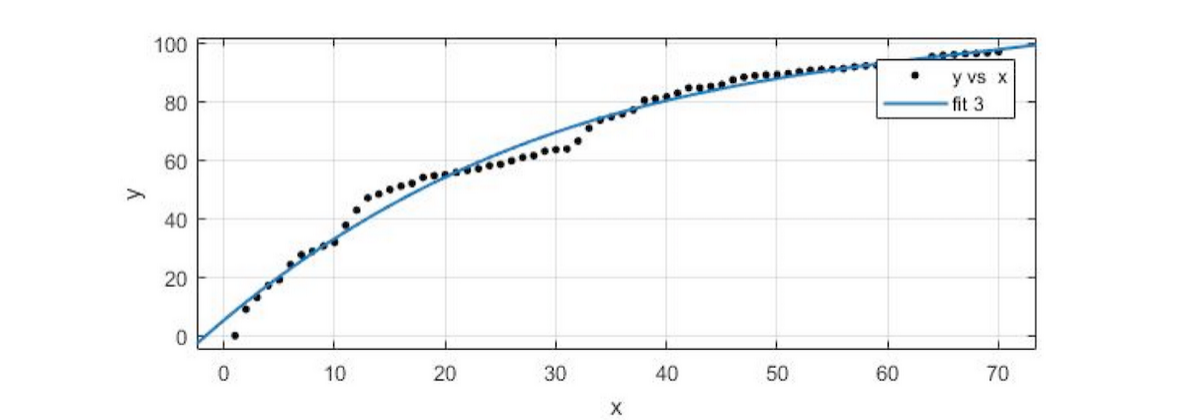
A data-fitting diagram of the zigzag-type incidents.

The third cluster, consisting of zigzag-type incidents, was also selected for the sample. For the forecast, we took the average data of the 5 incidents discussed in the Sina microblog and made use of the cftool toolkit in MATLAB R2017a to do the polynomial function fitting for prediction. As shown in Figure 5, *fit 3* was the fitting curve.

The fitting model of the polynomial function was

*f*(*x_3_*) = 0.0001973*x_3_*^3^−0.04018*x_3_*^2^ + 3.166*x_3_* + 5.273

where *x_3_* was the data observation point (we made an observation every 2 hours) and *f*(*x_3_*) was the value of the number of related tweets after standardized processing under the observation point.

In the fitting of the model, *R^2^* was 0.9872, the SSE was 584.7, adjusted *R^2^* was 0.9866, and RMSE was 2.977.

As shown in Table 4, the predicted data values were almost consistent with the actual values, with a maximum relative error of 4.441% and a minimum relative error of 0.8448%. The trend of the data in the forecast section (positions 71 to 84) completely coincided with the actual trend, which shows that the prediction effect of the model for the zigzag-type incidents was good.

**Table 4:** Comparison of the fitting value with the actual value of the zigzag-type incidents.

Predicted position	Fitting value	Actual value	Relative error (%)
71	98.1275	97.2985	0.8448
72	98.5737	97.5108	1.0783
73	99.0248	98.0012	1.0336
74	99.4820	98.2928	1.1954
75	99.9464	98.6039	1.3432
76	100.4193	98.8007	1.6118
77	100.9017	99.0006	1.8841
78	101.3950	99.1359	2.2280
79	101.9002	99.2251	2.6252
80	102.4186	99.2435	3.1001
81	102.9513	99.3665	3.4820
82	103.4996	99.6064	3.7616
83	104.0646	99.7724	4.1245
84	104.6474	100.0000	4.4410

#### Forecasting Trends in the Spread of Network Public Opinion: Steep Slope–Type Propagation

**Figure 6:**
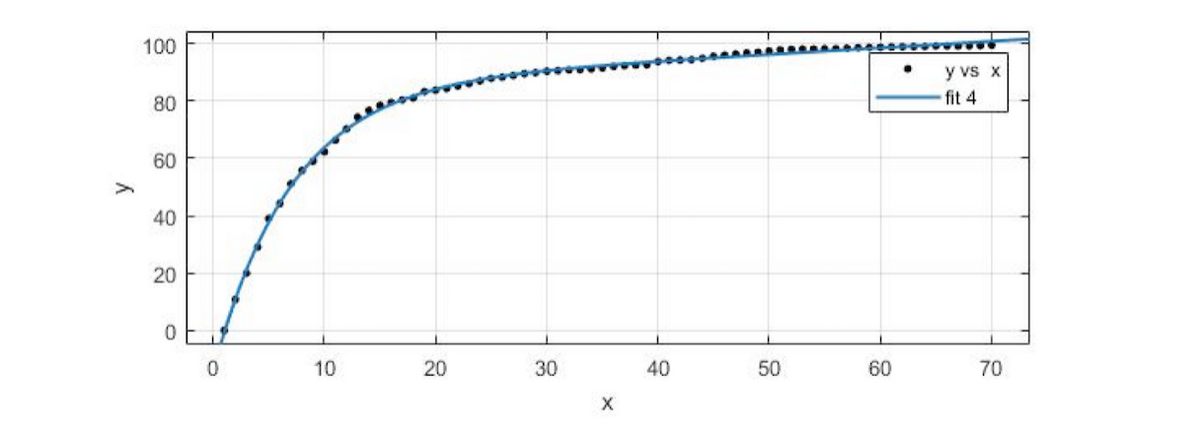
A data-fitting diagram of the steep slope–type incidents.

The seventh cluster, consisting of steep slope–type incidents, was selected. For the forecast, we took the average data of the 9 incidents in the Sina microblog and made use of the cftool toolkit in MATLAB R2017a to do the exponential function fitting for prediction. As shown in Figure 6, *fit 4* was the fitting curve.

The fitting model of the exponential function was

*f*(*x_4_*) = 85.79 × exp(0.002299 × *x_4_*)−100 × exp(−0.1424 × x_4_)

where *x_4_* was the data observation point (we made an observation every 2 hours) and *f*(*x_4_*) was the value of the number of related tweets after standardized processing under the observation point.

In the fitting of the model, *R^2^* was 0.9987, the SSE was 44.51, adjusted *R^2^* was 0.9986, and RMSE was 0.8212.

As shown in Table 5, the predicted data values were almost consistent with the actual values, with a maximum relative error of 3.9058% and a minimum relative error of 1.5555%. The trend of the data in the forecast section (positions 71 to 84) completely coincided with the actual trend, which shows that the prediction effect of the model for the steep slope–type incidents was good.

**Table 5:** A comparison of the fitting value with the actual value for the steep slope–type incidents.

Predicted position	Fitting value	Actual value	Relative error (%)
71	100.9970	99.4260341	1.5555
72	101.2300	99.4703353	1.7383
73	101.4635	99.5033538	1.9319
74	101.6975	99.5719260	2.0901
75	101.9319	99.5832087	2.3042
76	102.1668	99.6310762	2.4819
77	102.4022	99.6367044	2.7006
78	102.6382	99.6995552	2.8631
79	102.8746	99.7121893	3.0740
80	103.1116	99.7550284	3.2553
81	103.3490	99.8030223	3.4311
82	103.5870	99.8845621	3.5742
83	103.8256	99.9749575	3.7088
84	104.0646	100.0000000	3.9058

## Discussion

### Principal Findings

The theories and methods of public opinion communication are relatively mature, but applications in the field of medical public opinion are relatively few. We believe that this study is the first to apply the theories and methods of public opinion communication to research in medical public opinion and to systematically explore the types of incidents against medical staff and the patterns in which discussions about them propagate on microblogs. Tracking discussions of violent acts against medical staff in China that took place on the internet from 2011 to 2018, we selected 60 cases for our sample that prompted intense discussion on the Sina microblog, used crawler software to collect the data from the web, applied the SOM neural network model to cluster the tweets, mapped changes in the discussion trends over time, and used the polynomial and exponential function methods to analyze and predict communication patterns of public opinion. The results showed that the fit was good.

Our study found that the propagation of web-based public opinion relating to the violent incidents was divided into 8 types: bluff, waterfall, zigzag, steep, abrupt, wave, steep slope, and long slope. Over the first 12 hours or so, the number of tweets in the bluff type of discussion peaked, after which the growth rate declined suddenly in the 13th hour and continued to decline for 6 hours. In the 19th hour, there was an increase in the number of tweets that lasted for 6 hours or so, and then, the growth rate gradually decreased. After the 96th hour, the growth rate was almost 0. For the waterfall-type propagation, the early growth rate was slow. At about the 12th hour, the growth rate increased sharply, then gradually decreased. For the zigzag type of propagation, growth was rapid in the early stage, and then, it decreased and sometimes approached 0, going through a process of alternating slow and rapid growth. For the steep slope–type propagation, there was an early stage incubation period, then a peak period of posting from the 24th hour to the 48th hour, followed by gradually decreasing growth. After the 114th hour, the growth rate was almost 0. For the abrupt type, the growth rate was slow in the first 30 hours or so, and then, it increased slightly for about 2 hours. Before the 54th hour, the growth rate was almost flat, then it peaked between 54 and 72 hours and after that it gradually decreased. For the wave type, the number of tweets increased throughout the first 42 hours, then the growth rate showed a wave-shaped oscillation within 43 to 149 hours. After the 150th hour, the growth rate was almost 0. For the steep slope type, the number of tweets increased sharply over the first 6 hours or so, followed by a pause, and then by a gradual and steady increase from the 84th hour to 101st hour. After the 102nd hour, the growth rate was almost 0. For the long slope type, the growth rate of the tweets was stable and long lasting, persisting for 120 hours, after which it grew gradually and slowly.

What are the laws of propagation for the different types of violent medical incidents? We attempted to investigate this question with regard to the severity of consequences, the inducements for the incidents, and the popularity of the topic over the course of a week (in terms of the number of tweets). Our study found that the more serious the consequences of a violent incident, such as a serious injury or death, the more attention it drew on the microblog, the faster was its propagation speed, and the longer was its duration. In these cases, the propagation types were mostly of the steep slope, long slope, and zigzag types. In addition, the more serious the consequences of a violent incident, the greater popularity it exhibited on the microblog. Acts resulting from patients’ dissatisfaction with treatment effects led to significantly greater popularity within a week than incidents caused by nontherapeutic effects.

### Managerial Insights

Our research has important practical implications for both hospitals and governments. Workplace violence toward medical staff happens more often in China than in other countries, which provides multiple opportunities for research on the classification and prediction of incidents and their discussion on microblogs. The findings of this paper provide a new method for hospitals and health management departments in China and other countries to accurately predict communication types and rules of propagation in the discussion of violent incidents and can help relevant departments effectively manage public opinion concerning doctors and patients, ease workplace violence in the health sector, and improve doctor-patient relationships. Specifically, hospitals and health management departments should establish a public opinion monitoring system for doctor-patient relationships. When a violent incident occurs, relevant departments should pay close attention to the relevant tweets and comments and scientifically predict the communication types and rules of propagation based on the causes and severity of the consequences, so as to take effective countermeasures. In the case of incidents that lead to serious consequences, as these incidents would be quickly taken up in public discussion, hospitals and health management departments should promptly investigate the causes and announce the progress and results of the incidents. Some scholars emphasize the necessity to use both traditional media and social media in crisis response for mutual communication [10,32]. In the case of incidents that are caused by patients’ dissatisfaction with treatment effects, which the public often attributes to the low technical ability of medical staff and which easily generate heated discussions and lead to negative impacts on hospitals and staff, hospitals and health management departments should investigate the causes; take responsibility; and, through official channels and in a timely manner, clarify the situation, answer questions, and assuage doubts raised by the public.

### Limitations and Future Research

Our work has certain limitations that provide directions for future research. In this study, we explored the types and laws of the communication of events on the Sina microblog and analyzed the relationship among the types of communication, the laws of propagation, the causes of events, and the severity of the results. However, this study did not consider factors—such as the hospital type, the response strategies of health management departments and hospitals, and settlement results from judicial authorities—that could have an impact on the propagation of discussions of violent incidents on the microblog. We hope that future scholarly work, while attempting to confirm or refute our key findings, could also complement and expand the abovementioned issues. Future research is thus needed to select more incidents for the prediction analysis and improve the accuracy of the predictions.

## Appendix

Multimedia Appendix 1 Samples of violence against medical staff.

Multimedia Appendix 2 Multiple comparisons.

## Abbreviations

ANOVA: analysis of variance
RMSE: root-mean-square error
SOM: self-organizing map
SSE: sum of squares due to error
