# Supplementary material for: Classification and Prediction of Violence Against Chinese Medical Staff on the Sina Microblog Based on a Self-Organizing Map: Quantitative Study
Source: J Med Internet Res. 2020 May 26;22(5):e13294. doi: 10.2196/13294 (PMC7284412; doi:10.2196/13294)
Supplement: Multimedia Appendix 1 [file jmir_v22i5e13294_app1.docx]

**Table 1.** Samples of violence against medical staff.

| Location/date | Number of tweets (week) | Direct inducement | Order of severity |
| --- | --- | --- | --- |
| Nanjing Stomatological Hospital, February 25, 2014 | 10,988 | Privacy protection | Second-degree serious injury |
| Shiyan People’s Hospital, February 21, 2015 | 8232 | Treatment effect | Second-degree minor injury |
| Guangdong Provincial People’s Hospital, May 5, 2016 | 7568 | Patients’ or families’ own problems | Death |
| Wenling First People’s Hospital, October 25, 2013 | 6684 | Treatment effect | Death |
| Second Affiliated Hospital of Anhui Medical University, November 13, 2012 | 3322 | Treatment effect | Death |
| Chaozhou Central Hospital, March 5, 2014 | 2633 | Treatment effect | no bodily injury |
| Beigang Hospital, February 17, 2014 | 2348 | Treatment effect | Death |
| Hunan Academy of Traditional Chinese Medicine Affiliated Hospital, September 23, 2013 | 2009 | Treatment effect | Serious injury |
| First Teaching Hospital of Tianjin University of Traditional Chinese Medicine, November 29, 2012 | 1971 | Patients’ or families’ own problems | Death |
| Tongshan County People's Hospital, December 8, 2017 | 1737 | Treatment effect | Slight bodily injury (signs of abortion） |
| The Second Affiliated Hospital of Zhejiang University School of Medicine, February 20, 2014 | 1717 | Doctor-patient communication | Slight bodily injury (abortion） |
| First Affiliated Hospital of Harbin Medical University, March 23, 2012 | 1704 | Doctor-patient communication | Death |
| Ya’an People’s Hospital, January 5, 2017 | 851 | Doctor-patient communication | Slight bodily injury |
| Liaocheng People’s Hospital, December 4, 2017 | 753 | Treatment effect | Slight bodily injury |
| Tianjin Third Central Hospital, June 29, 2017 | 662 | Treatment effect | First-degree serious injury |
| Jiangsu Provincial People’s Hospital, February 16, 2017 | 653 | Patients’ or families’ own problems | Second-degree serious injury |
| Jingzhou First People’s Hospital, May 5, 2012 | 574 | Patients’ or families’ own problems | Slight bodily injury |
| Jixi People’s Hospital, July 11, 2017 | 473 | Doctor-patient communication | Serious influence on hospital order |
| Heping Hospital, affiliated with Changzhi Medical College, November 22, 2016 | 438 | Patients’ or families’ own problems | Serious injury |
| Dongguan Changping Hospital, April 17, 2016 | 426 | Response speed | Slight bodily injury |
| Huai'an First People's Hospital, February 10, 2018 | 406 | Response speed | Slight bodily injury |
| Gulin County People's Hospital, January 14, 2015 | 390 | Patients’ or families’ own problems | Slight bodily injury (abortion） |
| Qufu People’s Hospital, May 16, 2016 | 362 | Doctor-patient communication | Slight bodily injury |
| Changge People’s Hospital, November 1, 2015 | 358 | Patients’ or families’ own problems | Serious injury |
| Deyang People’s Hospital, January 3, 2016 | 339 | Response speed | Second-degree minor injury |
| Baoan District People’s Hospital, September 9, 2013 | 336 | Doctor-patient communication | Minor injury |
| Hong Lan Town Health Centre of Lishui District, April 23, 2017 | 322 | Treatment effect | Slight bodily injury |
| Shuyang County Nanguan Hospital, April 19, 2014 | 321 | Privacy protection | Second-degree minor injury |
| Huimin People's Hospital, June 15, 2017 | 290 | Treatment effect | Serious influence on hospital order and slight bodily injury |
| Jing’an District Central Hospital, March 4, 2016 | 288 | Patients’ or families’ own problems | Slight bodily injury |
| Shanxi Sengong Hospital, February 1, 2017 | 274 | Doctor-patient communication | Slight bodily injury |
| Yanminghu Central Health Center of Zhongmu County, June 11, 2015 | 270 | Patients’ or families’ own problems | Death |
| First Affiliated Hospital of Kunming Medical University, June 7, 2015 | 264 | Patients’ or families’ own problems | Serious injury |
| Women and Children Health Hospital of Duanzhou District, August 6, 2017 | 227 | Treatment effect | Slight bodily injury |
| Yibin Second People’s Hospital, December 17, 2017 | 221 | Treatment effect | Second-degree minor injury |
| Cixi People’s Hospital, June 4, 2017 | 206 | Treatment effect | Second-degree minor injury |
| Lai Feng County Central Hospital, November 2, 2017 | 193 | Treatment effect | Serious influence on hospital order |
| Du Ji People's Hospital, March 21, 2016 | 179 | Patients’ or families’ own problems | Death |
| Guangshui First People's Hospital, April 6, 2017 | 178 | Treatment effect | First-degree serious injury |
| Changxing County People's Hospital, October 4, 2016 | 173 | Patients’ or families’ own problems | Second-degree minor injury |
| Peking University Shenzhen Hospital, February 25, 2014 | 163 | Response speed | Slight bodily injury |
| Sichuan Orthopaedic Hospital, October 10, 2016 | 157 | Doctor-patient communication | Slight bodily injury |
| Ruijin Hospital, affiliated with the Medical College of Shanghai Jiaotong University, June 27, 2015 | 145 | Doctor-patient communication | Slight bodily injury |
| Orthopaedic Clinic in Jingjiang City, April 22, 2017 | 135 | Treatment effect | Death |
| Heilongjiang Provincial People's Hospital, August 15, 2017 | 122 | Treatment effect | Slight bodily injury |
| First People's Hospital of Chengdu, March 23, 2017 | 117 | Patients’ or families’ own problems | Slight bodily injury |
| Chengdu Infectious Disease Hospital, July 21, 2017 | 106 | Treatment effect | Second-degree minor injury |
| Lu'an People's Hospital, September 1, 2017 | 99 | Privacy protection | Second-degree minor injury |
| Taiyuan Central Hospital, August 5, 2017 | 94 | Patients’ or families’ own problems | Slight bodily injury |
| Shandong Wendeng Osteopathic Hospital, March 21, 2017 | 89 | Doctor-patient communication | Second-degree minor injury |
| Pengshui County People's Hospital, March 24, 2017 | 86 | Doctor-patient communication | Slight bodily injury |
| Nanjing General Hospital of Nanjing Command, January 17, 2017 | 86 | Patients’ or families’ own problems | Slight bodily injury |
| Jiangbei Town Center Hospital of Changsha County, May 17, 2016 | 86 | Doctor-patient communication | Minor injury |
| Fujian Provincial Cancer Hospital, February 7, 2017 | 83 | Treatment effect | Slight bodily injury |
| Sanhe People's Hospital, March 21, 2017 | 73 | Doctor-patient communication | Slight bodily injury |
| Shaodong County People's Hospital, July 17, 2017 | 70 | Response speed | Slight bodily injury |
| Traditional Chinese Medicine Hospital of Linshui County, August 5, 2017 | 57 | Doctor-patient communication | First-degree minor injury |
| Xinxiang Second People's Hospital, November 7, 2017 | 51 | Treatment effect | Serious influence on hospital order |
| Siping Central Hospital, October 15, 2017 | 50 | Patients’ or families’ own problems | Death |
| Zhoukou Central Hospital, July 12, 2017 | 49 | Patients’ or families’ own problems | First-degree minor injury |
