# Supplementary material for: Classification and Prediction of Violence Against Chinese Medical Staff on the Sina Microblog Based on a Self-Organizing Map: Quantitative Study
Source: J Med Internet Res. 2020 May 26;22(5):e13294. doi: 10.2196/13294 (PMC7284412; doi:10.2196/13294)
Supplement: Multimedia Appendix 2 [file jmir_v22i5e13294_app2.docx]

Table 3. Multiple comparisons.

| Medical incident and the types of propagation on the microblog (I) and (J) | | | Dependent variable: the severity of the consequences | | | |
| --- | --- | --- | --- | --- | --- | --- |
|  | | | Mean difference (I-J) | SE | Significance | 95% CI |
| **LSD (Least Significant Difference)** | | | | | | |
|  | **Bluff type** | | | | | |
|  |  | Waterfall type | −0.102 | 0.517 | .84 | −1.14 to 0.94 |
|  |  | Zigzag type | −1.175 | 0.634 | .07 | −2.45 to 0.10 |
|  |  | Steep type | 0.458 | 0.601 | .45 | −0.75 to 1.66 |
|  |  | Abrupt type | 0.125 | 0.681 | .85 | −1.24 to 1.49 |
|  |  | Wave type | 0.042 | 0.508 | .93 | −0.98 to 1.06 |
|  |  | Steep slope type | −*1.125^a^* | 0.556 | .05 | −2.24 to −0.01 |
|  |  | Long slope type | −*1.375^a^* | 0.634 | .03 | −2.65 to −0.10 |
|  | **Waterfall type** | | | | | |
|  |  | Bluff type | 0.102 | 0.517 | .84 | −0.94 to 1.14 |
|  |  | Zigzag type | −1.073 | 0.600 | .08 | −2.28 to 0.13 |
|  |  | Steep type | 0.561 | 0.565 | .32 | −0.57 to 1.69 |
|  |  | Abrupt type | 0.227 | 0.649 | .73 | −1.08 to 1.53 |
|  |  | Wave type | 0.144 | 0.464 | .76 | −0.79 to 1.08 |
|  |  | Steep slope type | −1.023 | 0.517 | .05 | −2.06 to 0.01 |
|  |  | Long slope type | −*1.273***^a^** | 0.600 | .04 | −2.48 to −0.07 |
|  | **Zigzag type** | | | | | |
|  |  | Bluff type | 1.175 | 0.634 | .07 | −0.10 to 2.45 |
|  |  | Waterfall type | 1.073 | 0.600 | .08 | −0.13 to 2.28 |
|  |  | Steep type | *1.633^a^* | 0.674 | .02 | 0.28 to 2.99 |
|  |  | Abrupt type | 1.300 | 0.746 | .09 | −0.20 to 2.80 |
|  |  | Wave type | *1.217***^a^** | 0.592 | .04 | 0.03 to 2.41 |
|  |  | Steep slope type | 0.050 | 0.634 | .94 | −1.22 to 1.32 |
|  |  | Long slope type | −0.200 | 0.704 | .78 | −1.61 to 1.21 |
|  | **Steep type** | | | | | |
|  |  | Bluff type | −0.458 | 0.601 | .45 | −1.66 to 0.75 |
|  |  | Waterfall type | −0.561 | 0.565 | .32 | −1.69 to 0.57 |
|  |  | Zigzag type | −*1.633^a^* | 0.674 | .02 | −2.99 to −0.28 |
|  |  | Abrupt type | −0.333 | 0.718 | .64 | −1.77 to 1.11 |
|  |  | Wave type | −0.417 | 0.556 | .46 | −1.53 to 0.70 |
|  |  | Steep slope type | −*1.583***^a^** | 0.601 | .01 | −2.79 to −0.38 |
|  |  | Long slope type | −*1.833***^a^** | 0.674 | .01 | −3.19 to −0.48 |
|  | **Abrupt type** | | | | | |
|  |  | Bluff type | −0.125 | 0.681 | .85 | −1.49 to 1.24 |
|  |  | Waterfall type | −0.227 | 0.649 | .73 | −1.53 to 1.08 |
|  |  | Zigzag type | −1.300 | 0.746 | .09 | −2.80 to 0.20 |
|  |  | Steep type | 0.333 | 0.718 | .64 | −1.11 to 1.77 |
|  |  | Wave type | −0.083 | 0.642 | .90 | −1.37 to 1.21 |
|  |  | Steep slope type | −1.250 | 0.681 | .07 | −2.62 to 0.12 |
|  |  | Long slope type | −1.500^a^ | 0.746 | .05 | −3.00 to 0.00 |
|  | **Wave type** | | | | | |
|  |  | Bluff type | −0.042 | 0.508 | .93 | −1.06 to 0.98 |
|  |  | Waterfall type | −0.144 | 0.464 | .76 | −1.08 to 0.79 |
|  |  | Zigzag type | −1.217^a^ | 0.592 | .04 | −2.41 to −0.03 |
|  |  | Steep type | 0.417 | 0.556 | .46 | −0.70 to 1.53 |
|  |  | Abrupt type | 0.083 | 0.642 | .90 | −1.21 to 1.37 |
|  |  | Steep slope type | −1.167^a^ | 0.508 | .03 | −2.19 to −0.15 |
|  |  | Long slope type | −1.417^a^ | 0.592 | .02 | −2.61 to −0.23 |
|  | **Steep slope type** | | | | | |
|  |  | Bluff type | *1.125***^a^** | 0.556 | .05 | 0.01 to 2.24 |
|  |  | Waterfall type | 1.023 | 0.517 | .05 | −0.01 to 2.06 |
|  |  | Zigzag type | −0.050 | 0.634 | .94 | −1.32 to 1.22 |
|  |  | Steep type | *1.583***^a^** | 0.601 | .01 | 0.38 to 2.79 |
|  |  | Abrupt type | 1.250 | 0.681 | .07 | −0.12 to 2.62 |
|  |  | Wave type | *1.167^a^* | 0.508 | .03 | 0.15 to 2.19 |
|  |  | Long slope type | −0.250 | 0.634 | .69 | −1.52 to 1.02 |
|  | **Long slope type** | | | | | |
|  |  | Bluff type | *1.375^a^* | 0.634 | .03 | 0.10 to 2.65 |
|  |  | Waterfall type | *1.273^a^* | 0.600 | .04 | 0.07 to 2.48 |
|  |  | Zigzag type | 0.200 | 0.704 | .78 | −1.21 to 1.61 |
|  |  | Steep type | *1.833^a^* | 0.674 | .01 | 0.48 to 3.19 |
|  |  | Abrupt type | *1.500^a^* | 0.746 | .05 | 0.00 to 3.00 |
|  |  | Wave type | *1.417^a^* | 0.592 | .02 | 0.23 to 2.61 |
|  |  | Steep slope type | 0.250 | 0.634 | .69 | −1.02 to 1.52 |
| **Tamhane** | | | | | | |
|  | **Bluff type** | | | | | |
|  |  | Waterfall type | −0.102 | 0.536 | 1.00 | −2.14 to 1.93 |
|  |  | Zigzag type | −1.175 | 0.719 | .99 | −4.46 to 2.11 |
|  |  | Steep type | 0.458 | 0.452 | 1.00 | −1.51 to 2.42 |
|  |  | Abrupt type | 0.125 | 0.510 | 1.00 | −2.02 to 2.27 |
|  |  | Wave type | 0.042 | 0.524 | 1.00 | −1.96 to 2.04 |
|  |  | Steep slope type | −1.125 | 0.557 | .84 | −3.27 to 1.02 |
|  |  | Long slope type | −1.375 | 0.881 | .99 | −5.88 to 3.13 |

^a^The mean difference is significant at the .05 level.
